# *Stenotrophomonas* sp. RZS 7, a novel PHB degrader isolated from plastic contaminated soil in Shahada, Maharashtra, Western India

**DOI:** 10.1007/s13205-016-0477-8

**Published:** 2016-08-24

**Authors:** S. J. Wani, S. S. Shaikh, B. Tabassum, R. Thakur, A. Gulati, R. Z. Sayyed

**Affiliations:** 1 Department of Microbiology, PSGVP Mandal’s, Arts, Science and Commerce College, Shahada, 425 409 Maharashtra India; 2 Microbial Prospection Division, CSIR-Institute of Himalayan Bioresource Technology, P.O. Box 6, Palampur, 176 061 Himachal Pardesh India; 3 Department of Zoology, Goverment Raza P G College, Rampur, 244 901 Uttar Pardesh India

**Keywords:** *Stenotrophomonas* sp., Poly-β-hydroxybutyrate, PHB depolymerase, Biodegradation

## Abstract

This paper reports an isolation and identification of novel poly-β-hydroxybutyrate (PHB) degrading bacterium *Stenotrophomonas* sp. RZS 7 and studies on its extracellular PHB degrading depolymerase enzyme. The bacterium isolated from soil samples of plastic contaminated sites of municipal area in Shahada, Maharashtra, Western India. It was identified as *Stenotrophomonas* sp. RZS 7 based on polyphasic approach. The bacterium grew well in minimal salt medium (MSM) and produced a zone (4.2 mm) of PHB hydrolysis on MSM containing PHB as the only source of nutrient. An optimum yield of enzyme was obtained on the fifth day of incubation at 37 °C and at pH 6.0. Further increase in enzyme production was recorded with Ca^2+^ ions, while other metal ions like Fe^2+^ (1 mM) and chemical viz. mercaptoethanol severally affected the production of enzyme.

## Introduction

Synthetic plastics create toxic pollution and many health hazards at every stage of their existence, i.e., right from their manufacturing, uses, recycling to the discarding. After their disposal, these synthetic plastics accumulate in the environment as recalcitrant. These environmental, health, and waste management concerns have led to the search of natural, safer, biodegradable, and an eco-friendly alternative to the hazardous synthetic polymers. In this regard, poly**-**β**-**hydroxybutyrate (PHB) have attracted commercial and research interest as a new biodegradable thermoplastic. PHB is accumulated as intracellular food storage by wide variety of microorganism under carbon rich growth conditions and is mobilized during nutrient starvation period under the influence of an enzyme PHB depolymerase (Klingbeil et al. 1996; Nojima 1996). PHB depolymerases hydrolyze PHB as a substrate and form monomers or dimers that serve as nutrients (Nakayama et al. 1985). The ability of microorganisms to degrade polyhydroxyalkanoates (PHAs) or PHB has been recorded from wide variety of Gram-positive and Gram-negative bacteria (Bradl and Puchner 1992; Mergaert et al. 1993, 1994, 1995) found in soil, compost, sewage etc. Since PHB has been widely accepted as biodegradable polymer, it is necessary to evaluate its biodegradation, the role of polymer-degrading microorganisms, factors affecting the activity of PHB degradation and kinetics of PHB degrading enzyme.

## Methods

### Sample collection

Soil samples were collected from plastic contaminated sites, dumping yards from municipal area location with latitude 21°30′47.09″N and altitude 74°28′40.47″E around Shahada, Maharashtra, Western India.

### Isolation and screening of PHB degrading bacteria

The PHB degrading bacteria were isolated and screened on minimal salt medium (MSM) containing PHB as the only carbon source (Tseng et al. 2007) at 28 ± 2 °C for 5–6 days and observed for presence of a zone of PHB hydrolysis around the colonies. Degree of PHB hydrolysis (zone of hydrolysis) was taken as a measure of PHB biodegradation. Colonies showing more zone of PHB hydrolysis were considered as potent PHB degrading isolates.

### Identification of potent PHB degrading strain

Potent PHB degrading isolate was identified using polyphasic approach. Physiological and biochemical characteristics were carried as per Bergey’s Manual of Systematic Bacteriology (Holt et al. 1994) using presterilized biochemical kits (Hi-Media, Mumbai, India). The ability of isolates to oxidize different carbon sources and production of various enzymes was investigated.

#### Identification by 16S rRNA

The sequencing of 16S rRNA genes of isolate was carried out as per the method describe by Gangurde et al. (2013). Genomic DNA of isolate was extracted by using HiPurA™ Plant Genomic DNA Miniprep Purification Spin Kit (HI-MEDIA, India) as per the method of Sambrook et al. (1989). Amplification of 16S rRNA gene sequencing was performed as per Pediyar et al. (2002) using the primers fD1 (5′-AGAGTTTG ATCCTGGCTCAG-3′) and rP2 (3′-ACGGCTACCTTGTTACGACTT-5′). The amplified sequences were analyzed with gapped BLASTn (http://www.ncbi.nlm.nih.gov), and the evolutionary distances were computed using the neighbor-joining method.

#### Identification by whole cell fatty acid methyl ester (FAME) analysis

The fatty acids from bacterial cultures grown on trypticase soya agar for 24 h at 28 °C were separated from cell and analyzed by gas chromatography (GC) (Agilent Technologies, USA) as per the method of Gulati et al. 2008. Fatty acids were identified and quantified by comparison to retention time and peak area obtained and compared with the fatty acid standards. Qualitative and quantitative differences in fatty acid profiles were used to compute the distance for each strain relative to other strains in the Sherlock bacterial fatty acid ITSA1 aerobe reference library (Sasser 2006).

#### BIOLOG identification

Utilization pattern for 95 carbon sources by isolate RZS 7 was studied using a BIOLOG system (Microstation, Microbial Identification System, 1998, Biolog Inc, CA, USA) as per the method of Shaikh et al. (2014). The development of color indicative of substrate utilization was read at 595 nm in a Micro Station Reader between 6 and 24 h of incubation. The substrate utilization profile was assessed with Micro Log version 4.2 database software.

### Production of PHB depolymerase from RZS 7

For production of PHB depolymerase, isolate RZS 7 (3 × 10^6^ cells/ml) was grown in MSM containing PHB, 0.15 %; K_2_HPO_4_, 0.7 g; KH_2_PO_4_, 0.7 g; MgSO_4_, 0.7 g; NH_4_Cl, 1.0 g; NaNO_3_, 1.0 g; NaCl, 5 mg; FeSO_4_, 2 mg, ZnSO_4_, 7 mg in 1 L of distilled water (Han and Kim 2002) at 120 rpm for 6 days at 30 °C. Enzyme activity was assayed after 24 h (1 day) interval.

#### PHB depolymerase assay

A uniform suspension of PHB granule in 50 mM Tris–HCl buffer (pH 7.0) was prepared by sonication at 20 kHz for 15 min, and 150 μg/ml of this PHB suspension and 2 mM CaCl_2_ and 100 μl of culture supernatant (source of PHB depolymerase) of RZS 7 were added to 50 mM Tris–HCl buffer (pH 7.0), and observed for decrease in turbidity of PHB preparation at 650 nm in spectrophotometer (Model 1240, Shimadzu, Japan). One unit of PHB depolymerase activity was defined as the quantity of enzyme required to decrease the OD (at 650 nm) by 0.1/min (Papaneophytou et al. 2009).

### Enzyme kinetic studies

#### Effect of temperature on enzyme activity

To ascertain temperature optima of enzyme, PHB depolymerase activity as described above was measured at different temperatures, i.e., 5, 27, 37, 45, 55, and 70 °C.

#### Effect of pH on enzyme activity

To determine the pH optima of enzyme, PHB depolymerase activity as described above was measured in buffers of varying pH in the range of 3.0–8.0.

#### Effect of metal ions on enzyme activity

Metal ions, such as Ca^2+^, Mg^2+^, Mn^2+^, Cu^2+^, Co^2+^, Hg^2+^, Zn^2+^, and Fe^2+^, were added at a concentration of 1 mM in the above mentioned reaction mixture and enzyme activity was measured as described earlier.

#### Effect of different chemicals on enzyme activity

Different chemicals, such as methanol (10 % v/v), ethanol (10 % v/v), acetone (10 % v/v), mercaptoethanol (1 % v/v), tween-20 (1 % v/v), tween-80 (1 % v/v), EDTA (1 mM), NaCl (1 mM), KCl (1 mM), and NaNO_3_ (1 mM), were individually added in the reaction mixture, and activity of PHB depolymerase was estimated as described earlier.

## Results and discussion

### Isolation and screening of PHB degrading strains

**Fig. 1:**
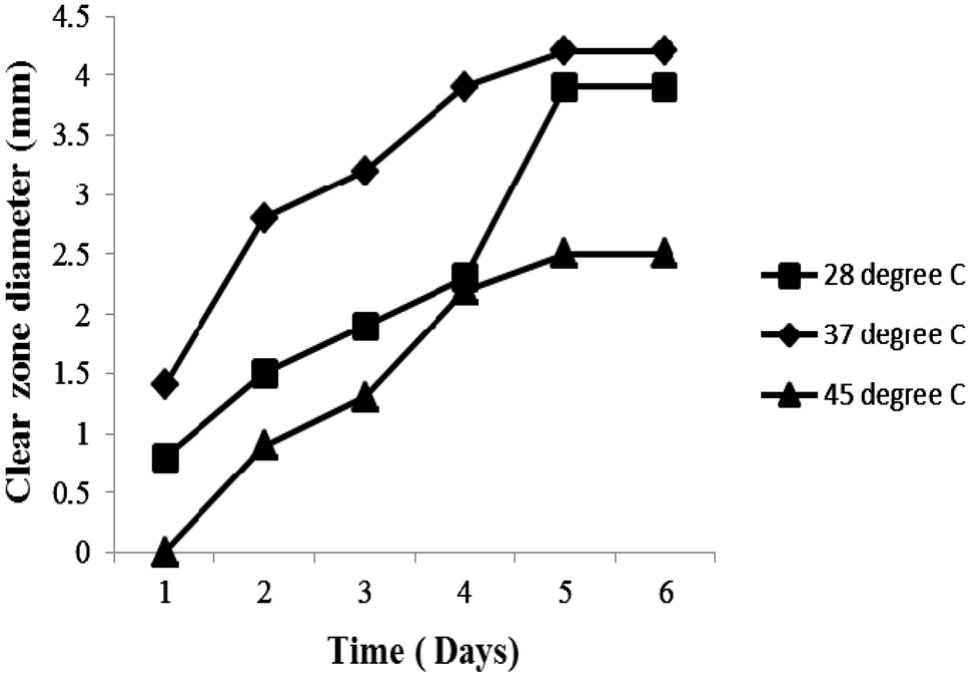
Time course of poly-β-hydroxybutyrate degradation as indicated by diameters (mm) of the clear zones on agar containing PHB at 28 °C, 37 and 45 °C

Among total 39 isolates obtained from representative plastic contaminated sites of municipal area of Shahada, Maharashtra, Western India, 6 isolates showed varying degree of PHB hydrolysis, and 1 isolate initially labeled as RZS7 showing maximum (4.2 mm) zone of PHB hydrolysis on MSM containing PHB that was considered as potent PHB degrader. This isolate started PHB degradation from the second day of incubation and resulted in maximum PHB degradation on the fifth day of incubation at 37 °C (Fig. 1).

### Identification of potent PHB degrading isolate

Preliminary phenotypic characterization of isolate showed that the PHB depolymerase producing isolate was Gram-negative, motile rod, and had fermentative metabolism of dextrose, L- arabinose, mannose, ribose, sucrose, lysine, ornithin, inulin, inositol, ONGP, and esculin. Preliminary phenotypic characterization identified the isolates as *Stenotrophomonas* sp.

#### Identification by 16S rRNA

The comparison of BLAST search of 16S rRNA gene sequences (1501 bp) of the isolate RZS 7 with 16S rRNA gene sequences of NCBI Gene Bank database showed the highest (99.32 %) identity of isolate to *Stenotrophomonas* *maltophilia*. Therefore, the isolate was identified as *Stenotrophomonas* sp. RZS 7. The 16S rRNA gene sequence of the isolate was submitted in NCBI Gene Bank under the name *Stenotrophomonas* sp. RZS 7 with accession No KP862608.

#### Identification by whole cell fatty acid methyl ester (FAME) analysis

The percentage and pattern of whole cell fatty acids of isolate RZS 7 was 11:0 iso (3.41 %), 11:0 anteiso (0.16 %), 10:0 2OH (0.21 %), 10:0 3OH (0.31 %), 11:0 iso 3OH (1.59 %), 13:0 iso (0.40 %), 12:0 iso 3OH (0.25 %), 12:0 3OH (3.69 %), 14:0 iso (0.88 %), 13:0 iso 3OH (2.84 %), 13:0 2OH (0.56 %), 15:1 iso F (0.89 %), 15:0 iso (29.67 %), 15:0 anteiso (12.71 %), 16:0 iso (1.56 %), 16:1 w9c (3.73 %), 17:0 iso (3.14 %), 17:0 anteiso (0.42 %), 17:1 w8c (0.27 %), 18:1 w9c (1.66 %), 19:0 iso (0.33 %), summed feature 3 (12.29 %), summed feature 8 (0.86 %), and summed feature 9 (3.54 %). These fatty acids and their distribution showed similarity index of 0.821 that resembled very well with *Stenotrophomonas* sp. (*Xanthomonas, Pseudomonas*).

#### BIOLOG identification

Carbon-source utilization pattern of isolate RZS 7 revealed growth on Dextrin, d-maltose, D-salicin, n-acetyl-D-glucosamine, n-acetyl-β-D-mannosamine, n-acetyl-D-galactosamine, NaCl 1 %, NaCl 4 %, d-fructose, sodium lactate 1 %, fusidic acid, d-serine, d-fructose-6-PO_4_, troleandomycin, rifamycin sv, gelatin, glycyl-l-proline, l-alanine, l-aspartic acid, l-glutamic acid, l-histidine, L-pyroglutamic acid, l-serine, lincomycin, guanidine HCl, niaproof 4, pectin, D-gluconic acid, glucuronamide, mucic acid, D-saccharic acid, vancomycin, tetrazolium violet, tetrazolium blue, p-hydroxy-phenylacetic acid, methyl pyruvate, L-lactic acid, citric acid, α-keto-glutaric acid, D-malic acid, L-malic acid, bromo-succinic acid, nalidixic acid, lithium chloride, tween 40, α-hydroxy-butyric acid, α-hydroxy-D butyric acid, α-keto-butyric acid, acetoacetic acid, propionic acid, acetic acid, formic acid, aztreonam, and sodium butyrate. The strain RZS 7 showed maximum (0.49) similarity index with *Stenotrophomonas* sp. in the BIOLOG database.

### Production of PHB depolymerase from RZS 7

PHB depolymerase activity of *Strenotrophomonas* RZS 7 after four days of incubation at 30 °C in MSM was 0.721 U/ml.

### Enzyme kinetic studies

#### Effect of temperature on enzyme activity

**Fig. 2:**
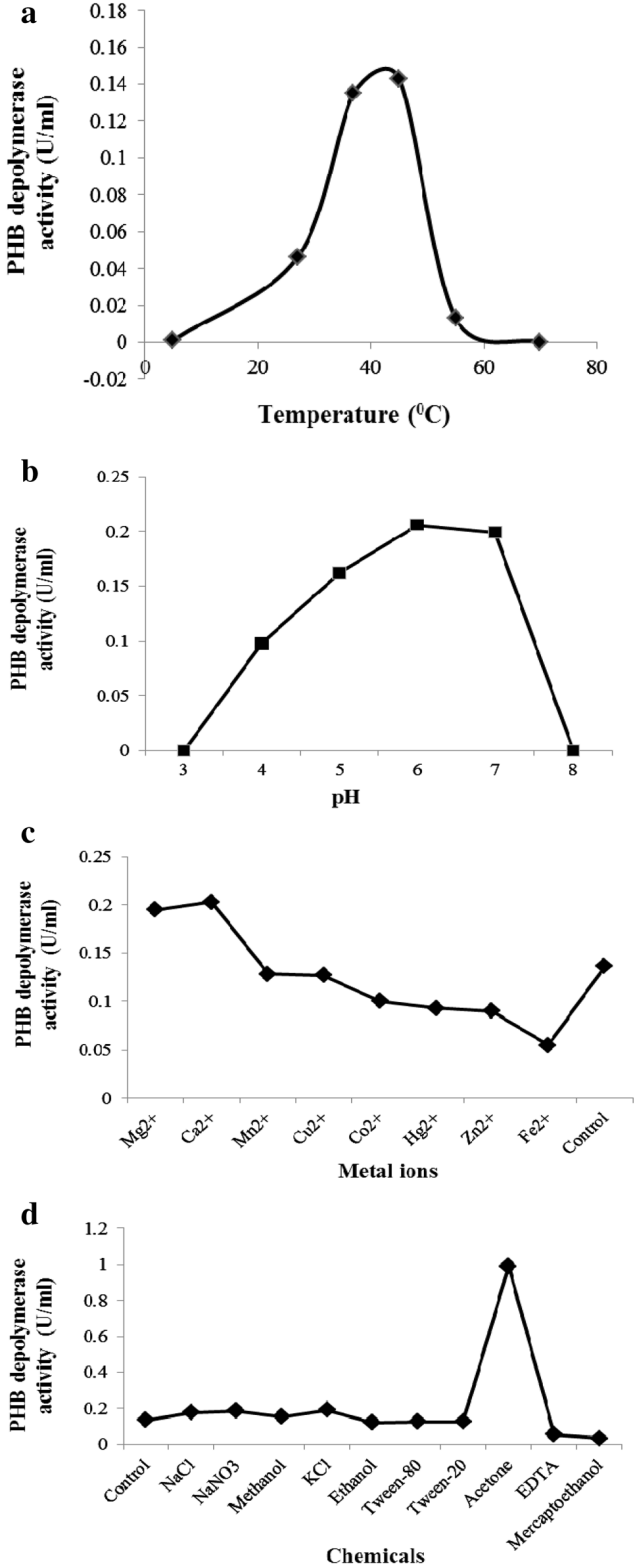
**a** Effect of temperature on poly-β-hydroxybutyrate depolymerase enzyme activity. **b** Effect of pH on poly-β-hydroxybutyrate depolymerase enzyme activity. **c** Effect of metal ions on poly-β-hydroxybutyrate depolymerase enzyme activity. **d** Effect of different chemicals on poly-β-hydroxybutyrate depolymerase enzyme activity

Optimum PHB depolymerase activity of *Stenotrophomonas* sp. RZS 7 was observed at 45 °C. The enzyme was thermally stable up to 45 °C, further increased in temperature above 45 °C, and decreased the enzyme activity; enzyme activity was completely inactivated at 70 °C (Fig. 2a). Thermostable PHB depolymerases have been reported from *Agrobacterium* and *Penicillium pinophilum* and some fungi (Nojima et al. 1996; Scherer 1996; Han et al. 1998).

#### Effect of pH on enzyme activity

Optimum PHB depolymerase activity of *Stenotrophomonas* sp. RZS 7 was observed at pH 6. The enzyme was stable in the acidic pH range between 4.0 and 7.0 (Fig. 2b). Sadocco et al. (1997) and Jeong (1996) have reported that the optimum pH of the PHB depolymerase isolated from bacteria was in the range of 7.0–9.0.

#### Effect of metal ions on enzyme activity

Among the various metal ions, Ca^2+^ ions served as activator of enzyme as it greatly enhanced the enzyme activity vis-à-vis Fe^2+^ ion that drastically affected the enzyme activity. Other metal ions, such as Mg^2+^, Mn^2+^, Cu^2+^, Co^2+^, Hg^2+^, and Zn^2+^, have no significant effect (Fig. 2c). Nojima et al. (1996) and Oda et al. (1997) have reported inhibitory action of metal ions on PHB depolymerase of *Agrobacterium* sp.

#### Effect of different chemicals on enzyme activity

Among the various chemicals evaluated for their effect on enzyme activity, mercaptoethanol caused maximum (85 %) inhibition of enzyme activity. Other chemicals, such as ethanol, ethanol, acetone, tween-20, tween-80, EDTA, NaCl, KCl, and NaN_3_, had no significant effect (Fig. 2d).

## Conclusion

A novel PHB depolymerase producing (PHB degrading) bacteria was isolated from plastic contaminated sites of Shahada, Maharashtra, Western India. It was identified as *Stenotrophomonas* sp. The PHB depolymerase of isolate was thermostable, acidophilic that required Ca^2+^ for its maximum activity. This study may help in tailoring the biodegradable and eco-friendly biopolymer for specific application. This, in turn, is expected to increase the commercial potential and use of this PHB as an eco-friendly and safer alternative to the hazardous synthetic polymer. This is the first report of degradation of PHB by depolymearse of *Stenotrophomonas* sp. RZS 7.
